# Glial-Restricted Precursors stimulate endogenous cytogenesis and effectively recover emotional deficits in a model of cytogenesis ablation

**DOI:** 10.21203/rs.3.rs-2747462/v1

**Published:** 2023-03-31

**Authors:** Luísa Pinto, Joana Macedo, Bruna Araújo, Sandra Anjo, Tiago Silveira-Rosa, Patrícia Patrício, Fábio Teixeira, Bruno Manadas, Ana Joao Rodrigues, Angelo Lepore, António Salgado, Eduardo Gomes

**Affiliations:** University of Minho; CNC - Center for Neuroscience and Cell Biology, University of Coimbra; University of Minho; University of Coimbra; University of Minho

## Abstract

Adult cytogenesis, the continuous generation of newly-born neurons (neurogenesis) and glial cells (gliogenesis) throughout life, is highly impaired in several neuropsychiatric disorders, such as Major Depressive Disorder (MDD), impacting negatively on cognitive and emotional domains. Despite playing a critical role in brain homeostasis, the importance of gliogenesis has been overlooked, both in healthy and diseased states. To examine the role of newly formed glia, we transplanted Glial Restricted Precursors (GRPs) into the adult hippocampal dentate gyrus (DG), or injected their secreted factors (secretome), into a previously validated transgenic GFAP-tk rat line, in which cytogenesis is transiently compromised. We explored the long-term effects of both treatments on physiological and behavioral outcomes. Grafted GRPs reversed anxiety-like and depressive-like deficits, while the secretome promoted recovery of only anxiety-like behavior. Furthermore, GRPs elicited a recovery of neurogenic and gliogenic levels in the ventral DG, highlighting the unique involvement of these cells in the regulation of brain cytogenesis. Both GRPs and their secretome induced significant alterations in the DG proteome, directly influencing proteins and pathways related to cytogenesis, regulation of neural plasticity and neuronal development. With this work, we demonstrate a valuable and specific contribution of glial progenitors to normalizing gliogenic levels, rescueing neurogenesis and, importantly, promoting recovery of emotional deficits characteristic of disorders such as MDD.

## Introduction

The adult mammalian central nervous system (CNS) can respond and adapt to environmental challenges, a phenomenon described as neural plasticity. Although this is still a provocative research topic, it has been reported that neural plasticity is strongly connected to cellular responsiveness and to the ability of the CNS to alter its function to a different demand, which is reflected in the capacity of restricted brain regions to persistently generate new neuronal (neurogenesis) and glial cells (gliogenesis) throughout life, a process collectively known as adult cytogenesis (1–5).

In the adult brain, the hippocampal dentate gyrus (DG) contains one of the main cytogenic niches and has been implicated in several mood, cognitive and emotional functions (6–9). Many of these functions are severely disturbed in conditions such as Major Depressive Disorder (MDD) (10), thus it is not surprising that adult cytogenesis and neural plasticity are highly compromised in MDD (11–14). Furthermore, treatment with antidepressants (ADs) is also known to modulate cytogenesis in the brain (15–18). In fact, several studies demonstrate that improvements in distinct behavioral components in rodent models of depression are accompanied by neuronal plasticity and neurogenesis recovery (19, 20). However, despite the compelling evidence that has elucidated the mechanisms of action of different classes of ADs, including their role in cytogenic processes, up to 30% of patients with MDD fail to respond to their first prescribed AD (21–23), and it is widely recognized that ADs present varying efficacies and numerous adverse effects (24–26). This major pitfall in the treatment of MDD and related conditions highlights the need to search for novel targets and develop new therapeutic approaches.

Considering that the generation and integration of new glial cells within the adult brain remains poorly understood, gliogenesis could represent an important area to be explored in depression-related research. Along these lines, it is already well documented that: i) human post-mortem samples from depressed patients (27, 28) and animal models of depression (herein reviewed (29)) present numerous alterations in the number, morphology and function of glial cells; ii) ADs impact distinct astroglial properties, namely their physiology, gene and protein expression, and even gliogenic levels (30–32); iii) astrocytes themselves can control adult hippocampal neurogenesis (33, 34); iv) recurrent stress exposure induces increased astrocytic density in the dorsal DG (dDG), which is prevented by treatment with ADs (35); and v) the AD imipramine acts as a pro-astrogliogenic factor and rescues cognitive impairments induced by stress exposure (36).

Nevertheless, glial cells and gliogenic processes still have not been incorporated as new therapeutic targets for treating neuropsychiatric disorders. Hence, improving the knowledge about glial cells as modulators of brain function, in physiological or diseased states, may allow us to better understand conditions such as MDD. One possible approach to achieve such a goal could be the transplantation of glial progenitors into models that present impairments of cytogenic processes. In this matter, our group has recently shed some light on the functional importance of newborn cells in specific developmental stages (37) through the use of the Glial Fibrillary Acidic Protein (GFAP)-thymidine kinase (tk) rat model (38), in which newborn GFAP-positive cells are selectively ablated for a transient time. We demonstrated that GFAP-tk rats that lack newborn immature hippocampal neuronal and glial cells present hyperanxiety and cognitive rigidity, while specific abrogation of mature cells resulted in long-term alterations in anxiety and hedonic behaviors, along with deficits in multiple cognitive modalities, which could be related to impairments in the communication between the hippocampus and the medial prefrontal cortex (37).

Building on this knowledge, in the present study we transplanted Glial Restricted Precursors (GRPs) or their secreted factors (secretome) into the dDG of GFAP-tk rats, with the objective of boosting gliogenesis, and assessing their effects both on endogenous cytogenesis and on different behavioral outcomes that are disturbed in this animal model. GRPs are progenitor cells restricted to the glial lineage (39–41), namely astrocytes and oligodendrocytes, and may be a powerful tool to use as a new platform to develop novel therapeutic targets based on glial cell development and modulation. Our results highlight the potential of modulating gliogenesis and glial plasticity as key factors to revert deficits related to cytogenesis impairment and prompt the field to further explore the role of new cells in brain networks and function, which are compromised in diseased states.

## Methods

### GRP derivation and culture in vitro

GRPs were derived from the spinal cord of E13.5 embryos of Sprague-Dawley rats that transgenically express the human alkaline phosphatase (APP) gene. The spinal cords were isolated from embryos and the meninges were removed. Then, the spinal cords were dissociated into single-cell suspensions with Neural Tissue Dissociation Kits (Miltenyi Biotec) (42). After obtaining the cells, GRPs were firstly thawed and seeded onto a T75 flask (3.5 × 10^4^ cells/cm^2^), previously coated with 15 μg/ml of Poly-L-Lysine (Sigma) and 10 μg/mL of Laminin (Sigma). Then, these cells were cultured in GRP medium, until further experimental procedures: DMEM/F12 (Thermo Fisher Scientific), 1% of PenStrep (Thermo Fisher Scientific), BSA (1 mg/ml; Sigma), B-27 Serum Free Supplement (1×, Thermo Fisher Scientific), bFGF without Vitamin A (10 ng/ml; PrepoTech) and N2 Supplement (1×, Thermo Fisher Scientific). GRPs were characterized at P5 as described in the Supplementary Methods.

### GRP secretome collection

For GRP secretome harvest, defined as conditioned media (CM), GRPs at cell passage (P5) were cultured until 80% confluence in GRP medium, washed one time with DMEM/F12 and cultured in basal media only for 8h. Collected CM was centrifuged at 300×g for 5 min to remove cell debris. Then, the CM was concentrated using centrifugal filter units with a 5-kDA cutoff, (Vivaspin^®^, GE Healthcare) at 3.000×g, at 4°C and stored at − 80°C for further analysis and experiments.

### Animal Model

To selectively ablate neural stem cells, we used a transgenic GFAP-tk rat model (38). In the GFAP-tk model, viral thymidine kinase (tk) is expressed under the GFAP promoter. Hence, upon treatment with the anti-viral agent ganciclovir (GCV), the dividing GFAP-expressing neural stem cells are eliminated(37, 38).

Three weeks after GFAP-tk transgenic rats and their wild-type (WT) littermate controls were born, weaning was performed, where males were separated from females, and grouped-housed in polypropylene cages (two per cage) to promote socialization, under 12h light – 12h dark cycles, 22°C, relative humidity of 55% and with food and water ad *libitum*. Furthermore, their identification was made by ear punch, in the left ear, to one animal of each cage. Ear biopsy was done to all the animals, for genotyping.

All procedures were carried out in accordance with EU Directive 2010/63/EU guidelines on animal care and experimentation and were approved by the University of Minho Subcommittee of Ethics for the Life and Health Sciences and by the Portuguese National Authority for animal experimentation, Direção-Geral de Alimentação e Veterinária (DGAV 014085).

All GFAP-tk transgenic rats and WT littermate controls used in this study were Sprague-Dawley rats (n = 40 animals: 29 GFAP-tk and 11 WT), and the treatment with GCV was initiated at 12 weeks of age, for a period of 18 days [20 mg/Kg were injected intraperitoneally (i.p.), once a day]. Surgical interventions were done 12 days after the end of the treatment with GCV.

### Grp Transplantation/secretome Injection And Post-operative Care

Rats were anesthetized with a combination of medetomidine hydrochloride (i.p.; Domitor; 0.5 mg/Kg, Pfizer) and ketamine (i.p.; Imalgene; 75 mg/Kg, Merial). Then, they were placed in a stereotactic frame and the skull was bilaterally injected with 2 μL of GRPs (5.0 × 10^4^ cells/μL) or GRP secretome, using a Hamilton 30-gauge syringe into the hippocampal DG, with the following brain coordinates: dorsal-ventral: −4.4 to −3.4, lateral-medial: ±1.6, and anterior-posterior: −3.3, according to Paxinos and Watson, 2006 (43). Sham animals were used as a control, being subjected to an injection without content in both left and right DG. The animals were divided into 3 groups: injected with GRPs, GRP secretome, and sham (without injection content). The injection time was 10 min, and after the injection the needle was left *in situ* for 5 min, being slowly removed afterwards. All animals were sutured with sterile 3 − 0 silk sutures and injected subcutaneously with buprenorphine (Bupaq; Analgesic; 0.05 mg/Kg, Richter Pharma), carprofen (Rimadyl; Anti-inflammatory; 5 mg/Kg, Pfizer) and atipamezole (Antisedan; Anesthesia Reverser; 1 mg/Kg, Pfizer). Animals were allowed to recover in a cage with a heating lamp and monitored every hour for the first 6h. Then, they returned to their home cage and were monitored every 12h, during the first 3 days after surgery.

### Behavioral Tests

The behavioral impact of GRP transplantation and their secretome into the adult hippocampal DG was assessed through different assays. Novelty Suppressed Feeding (NSF) and Elevated Plus Maze (EPM) tests were implemented to assess anxiety-like traits. A Forced Swim Test (FST) was conducted to assess depressive-like behavior. These tests were executed four weeks after surgeries, with the intent of observing a re-establishment of the neurogenic pool in a long-term period and assessing behavior modulation. Detailed description of the behavioral tests is provided in the Supplementary Methods.

### Brdu Labeling

The proliferation and maturation of new cells in the DG were assessed using an exogenous cell tracer 5’-Bromo-2’-Deoxyuridine (BrdU, Sigma-Aldrich) – a thymidine analog that is incorporated into the DNA during the S-phase of the cell cycle – together with endogenous neural cell markers. To detect newborn cells, all animals received a single i.p. injection of 50 mg/Kg BrdU dissolved in 0.9% of saline solution, for five uninterrupted days, one week after surgeries.

### Immunohistochemistry

Cryostat sections were incubated in PFA 4% for 30 min and washed (3 × 5 min) in PBS 1×. Antigen retrieval was then performed for 20 min, through heated citrate buffer. Then, slides were washed with 0.5% Triton X-100 (3 × 5 minutes) following an incubation step with the same solution for 10 min, and after, the sections were incubated in hydrochloric acid 2N for 30 min at RT. This step was performed only for specific stainings, where double staining with BrdU was implemented (the double staining BrdU/APP was an exception). Following this step, sections were washed again with 0.5% Triton X-100 (3 × 5 min) and incubated in blocking serum [10% normal goat serum (NGS), Abcam] for 30 min. Subsequently, tissue sections were incubated with primary antibodies, followed by secondary antibodies as described in detail in the Supplementary Methods.

### Mass Spectrometry Analysis

Secretome samples were analyzed on a NanoLC^™^ 425 System coupled to a Triple TOF^™^ 6600 mass spectrometer (Sciex^®^) and the DG samples were analyzed on a nanoLC Ultra 2D system (Eksigent^®^) coupled to a Triple TOF^™^ 6600 System (AB Sciex^®^) using two acquisition modes: i) the pooled samples were analyzed by IDA and, ii) the individual samples by SWATH-MS mode. Detailed description of the protein extraction and digestion and Mass spectrometry procedure is provided in the Supplementary Methods.

### Gene Ontology and functional analyses

Gene ontology (GO) enrichment analyses, considering REACTOME pathways or biological process categories, in the case of the tissue proteomics or the secretome characterization, respectively, were performed using David Bioinformatics Resources (54) with the statistical Fisher exact test associated. Different p-values were used as cut-off depending on the comparison (see supplementary tables S1 and S2). REVIGO (http://revigo.irb.hr/) (55) was used to remove the redundancy obtained in the analysis of the biological processes. Functional protein association networks were evaluated using the Search Tool for Retrieval of Interacting Genes/Proteins (STRING) 11.5 (http://string-db.org/) (56) with medium confidence (0.4) parameters.

The GO Enrichment Plot function in ImageGP (http://www.ehbio.com/ImageGP/) (57) was used to integrate and represent the Gene Ontology analysis. The heatmaps from the quantified proteins were performed in Morpheus (https://software.broadinstitute.org/morpheus/). Each row corresponds to a different protein, with their relative levels normalized to values between 0 and 1.

### Statistical analysis

Statistical evaluation was performed using IBM SPSS Statistics ver.22 (IBM Co.), and graphical representation was performed using GraphPad Prism ver.6 (GraphPad Software). Statistical analysis for behavioral tests was performed using one-way ANOVA to compare the mean values between groups. Normality was measured using the Kolmogorov-Smirnov and Shapiro-Wilk statistical tests, taking into account the respective histograms and measures of skewness and kurtosis. Equality of variances and Sphericity were measured using the Levene’s and Mauchly’s tests, respectively, and was assumed when p > 0.05. Multiple comparisons between groups were accomplished through the Tukey statistical test. Values were accepted as significant if the p-value was lower than 0.05 and all results were expressed as group mean ± SEM (standard error of the mean), with a 95% confidence interval. To identify the proteins differentially regulated, a Kruskal–Wallis H test was performed followed by the Dunn’s test for pairwise comparisons. Dunn’s p-values were corrected using the Benjamini–Hochberg FDR adjustment and statistical significance was considered for corrected p-values below 0.05. All the analyses were performed using the normalized protein levels in R version 3.5.2 using the test function available in the native stats package in R.

## Results

### GRPs maintain a progenitor phenotype both in vitro and in vivo, and are mainly restricted to the glial lineage

**Figure 1:**
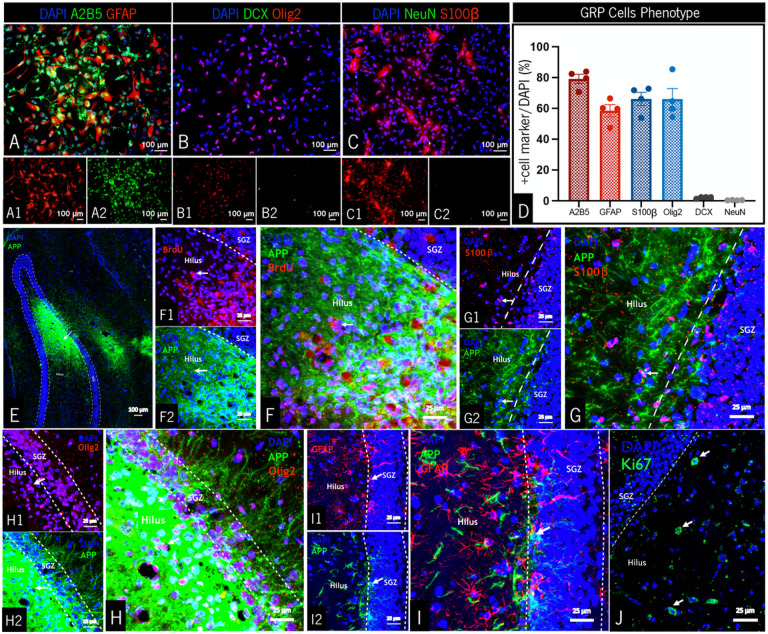
Characterization of GRPs at passage 5, both *in vitro* and *in vivo* (post transplantation). Immunocytochemistry for GFAP (**A1**)/A2B5 (**A2**), Olig2 (**B1**)/DCX (**B2**) and S100β (**C1**)/ NeuN (**C2**) markers, and their respective quantification (**D**). GRPs maintained an overall progenitor phenotype, mainly differentiating into glial cells. (**E**) Representative brain section of a WT rat transplanted with GRPs, with most of the cells primarily concentrated in the dDG. (**F**) BrdU positive cells, within the hilar region of the dDG, co-localized (white arrows) with APP positive cells (GRPs), confirming that transplanted cells were proliferating one week post grafting. Approximately 40 days after transplantation, GRPs differentiated into mature astrocytes (S100β+, white arrows in **G**), oligodendrocytes (Olig2+, white arrows in **H**) and immature astrocytes (GFAP+, white arrows in **I**), highlighting the glial differentiation pattern of these cells. (**J**) Proliferating cells (Ki67+, white arrows) were still found within the dDG, 40 days after GRPs transplantation. Data is presented as Mean ± SEM. Scale (1A-1E): 100 μm; scale (1F-1J): 25 μm.

To characterize the population of glial progenitors under study, GRPs were cultured *in vitro* until passage five (P5, the passage used in all experiments) and immunostained for various neuronal and glial lineage markers. Five days post-seeding, a high percentage of cells expressed the glial progenitor marker A2B5 (Figs. 1A/1D, $\bar{x}=79.14\%$), as well as the astrocyte/neural stem cell marker GFAP (Figs. 1A/1D, $\bar{x}=58.44\%$). GRPs also expressed glial cell differentiation markers, presenting positive staining for Olig2 (oligodendrocytes, Figs. 1B/1D, $\bar{x}=64.66\%$) and S100β (mature astrocytes, Figs. 1C/1D, $\bar{x}=66.19\%$). Regarding GRP differentiation into the neuronal lineage, markers for immature (DCX, Figs. 1B/1D, $\bar{x}=2.40\%$) and mature (NeuN, Figs. 1C/1D, $\bar{x}=0.57\%$) neurons were observed at only a negligible percentage.

Regarding the integration of exogenous glial progenitors in an *in vivo* scenario, GRPs (P5) were transplanted to the dDG of WT sham rats, with the objective of assessing cellular survival and differentiation patterns. Following a period of approximately 40 days post-transplantation, brain tissue sections revealed high levels of APP positive staining (GRP specific marker), which indicates that transplanted cells survived and were dispersed not only along the dDG, but also in the surrounding areas (Fig. 1E). Moreover, after labeling proliferating cells through BrdU injections (seven days post-GRP transplantation), we observed BrdU+/APP + double staining (Fig. 1F), confirming that GRPs were able to proliferate within the host cellular environment. Furthermore, similarly to what was performed in the *in vitro* cultures, GRPs were assessed for the expression of differentiation markers. Transplanted cells (APP+) presented positive staining for both S100β (Fig. 1G), Olig2 (Fig. 1H) and GFAP (Fig. 1I), confirming that GRPs differentiated *in vivo* into (mature) astrocytes (S100β- and GFAP-positive) and oligodendrocytes (Olig2-positive). In this same time point, and as expected, proliferating cells (Ki67+) were detected (Fig. 1J), obviating deleterious effect of GRP transplants on hippocampal cellular proliferation.

### GFAP-tk rat model characterization

**Figure 2:**
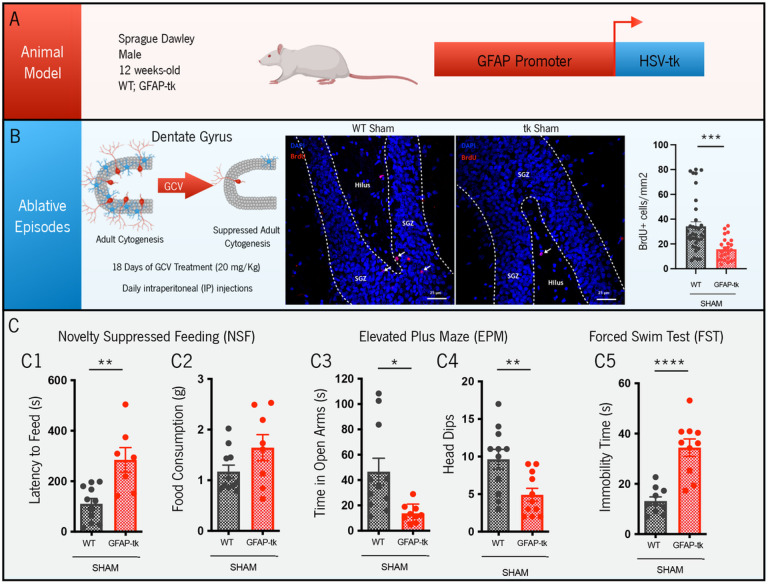
GFAP-tk rat model characterization. **(A)**In this study, 12 weeks-old Sprague Dawley transgenic male rats were used, expressing the viral HSV-tk under the GFAP promoter, with their WT littermates being used as controls. **(B)** Both groups were treated with the anti-viral GCV for 18 days, leading to suppression of adult cytogenesis in GFAP-tk animals (fewer BrdU+ cells in the DG). Afterwards, due to this impaired cytogenic process, transgenic animals revealed anxiety-like behavior in the NSF (**C1**) and EPM tests (**C3-C4**), while maintaining a normal food consumption pattern (**C2**). GFAP-tk rats further presented a depressive-like phenotype in the FST (C5). Data is presented as Mean ± SEM. *p<0.05, **p<0.01, ***p<0.001, ****p<0.0001.

As detailed in the Methods section, to induce cytogenesis ablation, we treated male Sprague Dawley transgenic GFAP-tk rats (using their WT littermates as controls) for 18 days (Fig. 2A/2B) with the anti-viral agent GCV. Corroborating previous results (37), we verified that GFAP-tk sham animals presented a significant reduction in the number of BrdU + cells, when compared to WT controls, as represented in Fig. 2B (t_58_ = 3.893, p < 0.001). This result clearly indicates the impaired cytogenic process in these transgenic animals. In addition, to validate the impact of this alteration at the behavioral level, we performed anxiety-like (NSF and EPM) and depressive-like (FST) behavioral tests. In the NSF, GFAP-tk sham rats presented an increased latency to feed, when compared to WT sham rats (Fig. 2C1, t_16_ = 3.739, p = 0.0018), while maintaining normal levels of food consumption (Fig. 2C2, t_17_ = 1.787, p = 0.0917) and normal weight variation (Fig. S1), which reveals an anxiety-like phenotype in those animals. In the same direction, in the EPM, GFAP-tk rats spent less time in the open arms of the EPM apparatus (Fig. 2C3, t_18_ = 2.772, p = 0.0126) and performed fewer head dips (Fig. 2C4, t_20_ = 2.987, p = 0.0073), when compared to controls. In the FST, transgenic rats presented higher immobility times (Fig. 2C5, t_18_ = 5.551, p < 0.0001), in comparison to WT sham animals, which suggests a depressive-like pattern. Altogether, it was possible to validate this GFAP-tk model in terms of cellular alterations (ablation of newly born cells) and anxiety-like and depressive-like behaviors.

### GRP transplants can revert both behavioral deficits and cytogenic impairments induced by the ablation of endogenous newly born cells

**Figure 3:**
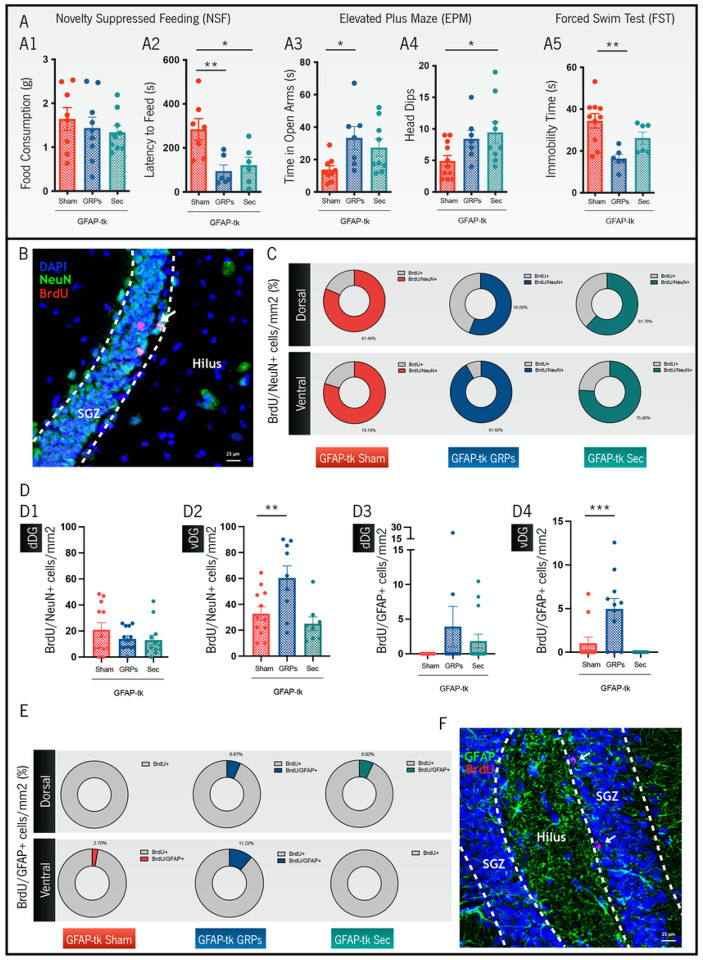
Effects of GRP and secretome treatments on behavior and generation of new cells in GFAP-tk rats. (**A**) Anxiety- and depressive-like behavioral characterization in GFAP-tk animals through the NSF (**A1-A2**), EPM (**A3-A4**), and FST tests (**A5**). Both treatments with GRPs and their secretome induced a recovery in anxiety-like behavior, while GRPs were also capable of reverting depressive-like behavior. (**B-F**) Evaluation of the cytogenic potential in GFAP-tk animals. (**B**) Representative microphotograph of BrdU+ cells co-localizing with NeuN+ cells in the SGZ of the dDG, in a GFAP-tk rat treated with GRPs. (**C**) Percentage of cells co-localizing BrdU/NeuN markers, from the total BrdU+ cells, for all groups in the study and for the dDG and vDG regions. (**D**) Quantification of the total number of cells co-localizing BrdU/NeuN markers, for all groups in the study and for the dDG (**D1**) and vDG (**D2**) regions. (**E**) Percentage of cells co-localizing BrdU/GFAP markers, from the total BrdU+ cells, for all groups in the study and for the dDG and vDG regions. (**D**) Quantification of the total number of cells co-localizing BrdU/GFAP markers, for all groups in the study and for the dDG (**D3**) and vDG (**D4**) regions. (**F**) Representative microphotograph of BrdU+ cells co-localizing with GFAP+ cells in the SGZ of the dDG, in a GFAP-tk rat treated with GRPs. Data is presented as Mean ± SEM. *p<0.05, **p<0.01, ***p<0.001.

sTwelve days after inducing cytogenesis ablation, we administrated GRPs or their secretome into the dDG of GFAP-tk animals. Following approximately four weeks, we explored if transplanted cells and/or their secretome were able to revert the behavioral deficits observed in this animal model. In the NSF test, we firstly confirmed that treated animals (either with GRPs or secretome) showed no differences regarding food consumption when compared to GFAP-tk sham rats (Fig. 3A1, F_(2,23)_ = 0.4773, p = 0.6265). However, as significant differences among groups were observed in the latency to feed (Fig. 3A2, F_(2,16)_ = 6.965, p = 0.0067), we verified that both secretome-treated animals (Fig. 3A2, p = 0.0256) and GRP-treated animals (Fig. 3A2, p = 0.0095) presented a reduced latency to feed, when compared to GFAP-tk sham rats. This result highlights that GRP and secretome treatments were able to induce recovery of the anxiety-like behavioral phenotype. We further complemented this finding by evaluating anxiety-like behavior through the EPM test, revealing that rats transplanted with GRPs spent more time exploring the open arms of the apparatus in comparison to GFAP-tk sham animals (Fig. 3A3, p = 0.0312), while secretome-treated rats performed more head dips when compared to the same transgenic animals (Fig. 3A4, p = 0.0412). These findings indicate that both treatments have a positive effect on anxiety-like behaviors. Regarding depressive-like behavior, and following the FST test, we registered behavioral differences among groups (Fig. 3A5, F_(2,19)_ = 8.085, p = 0.0029), verifying that GRP-treated rats showed a significant reduction in the immobility time when compared to GFAP-tk sham animals (Fig. 3A5, p = 0.0021), pointing out a positive effect in this particular behavioral domain. Overall, we show that it was possible to revert depressive-like and anxiety-like behaviors through the transplantation of GRPs into the dDG of GFAP-tk rats, while the injection of GRP secretome was capable of reverting anxiety-like traits in the same animal model.

Subsequently, approximately 40 days after grafting GRPs or injecting their secretome, we analyzed the brain tissues from these animals, in particular the DG, in order to explore cellular proliferation patterns. Specifically, we observed that all groups presented proliferating cells that differentiated into mature neurons (BrdU+/NeuN + cells, Fig. 3B–C), both in the dDG and in the vDG. Notably, in the latter, there was a significant difference between groups (Fig. 3D2, F_(2,26)_ = 7.115, p = 0.003), namely, an increased number of BrdU+/NeuN + cells in GFAP-tk animals transplanted with GRPs when compared to GFAP-tk sham rats (Fig. 3D2, p = 0.01). Moreover, we also searched for proliferating glial/neural stem cells and observed only a few BrdU+/GFAP + cells in all groups (Fig. 3E–3F), with no significant differences among them, in the dDG (Fig. 3D3). Interestingly, in the vDG we found differences among groups (Fig. 3D4, F_(2,29)_ = 9.875, p < 0.001), and GFAP-tk animals transplanted with GRPs significantly presented more BrdU+/GFAP + cells when compared to the GFAP-tk sham rats (Fig. 3D4, p = 0.004).

### GRPs and secretome treatments alter the proteomic profile of GFAP-tk rats’ DG

**Figure 4:**
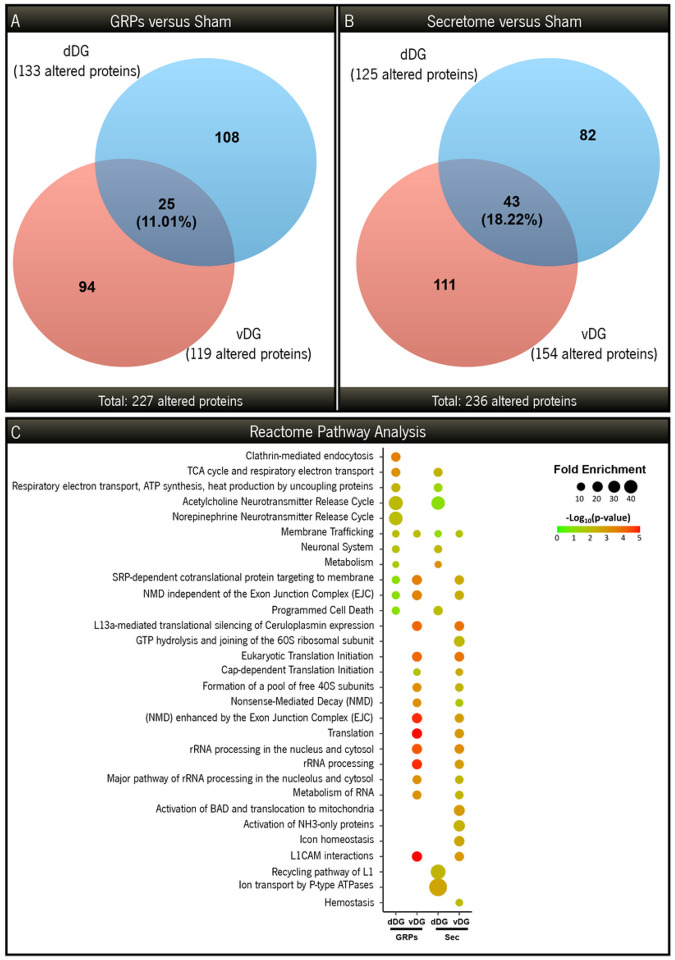
Analysis of the proteins significantly altered between GRPs or Secretome treatments and GFAP-tk sham animals. Venn diagrams representing the total number of proteins significantly altered between GRPs-treated (**A**) or Secretome-treated rats (**B**) and GFAP-tk sham animals, both for the dDG (blue) and vDG regions (red). (**C**) Comparative pathway analysis covering the most significant and enriched Reactome pathways, taking into consideration the proteins uniquely altered in each region (dDG and vDG) between GRP-treated or secretome-treated animals and GFAP-tk sham rats.

In an attempt to assess the biological effects of GRP transplantation or their secretome injection in the GFAP-tk rat model, we collected both the dDG and the vDG for proteomic analysis. By analyzing relative expression levels, we have found a total of 227 differentially expressed proteins between GRP-treated and GFAP-tk sham animals (Fig. 4A), while secretome administration resulted in a total of 236 differentially expressed proteins, in comparison to GFAP-tk sham rats (Fig. 4B). Overall, only 11.01% (in GRP-treated group) and 18.22% (in secretome-treated animals) of the altered proteins were common to both regions analyzed, which highlights a region-specific response to each treatment. An unbiased reactome pathway analysis revealed several enriched biological pathways in both treated groups (Fig. 4C). In the dDG, the pathways related to energy production and metabolism, and importantly, membrane trafficking and neurotransmitter release, were enriched, in particular in GRP-treated animals (Fig. 4C). With a very distinct profile, the vDG presented several pathways related to protein synthesis and processing, once again with higher relevance in GRP-treated rats (Fig. 4C). Interestingly, there was also an enrichment of the L1CAM interactions pathway, a family of proteins predominantly expressed in neurons during development.

Using the same sets of differentially expressed proteins, we also performed a STRING analysis focused on biological processes, dividing the results by region for each treatment (Fig. S2). We considered three main clusters for each region analyzed, and for all regions, there was at least one cluster of proteins related to neuronal processes. Hence, these results show that both GRPs (Fig. S2, A1-A2) and their secretome (Fig. S2, B1-B2) influenced processes such as neurotransmitter signaling, synaptic plasticity, neurogenesis, and neuronal differentiation. Overall, there was an effect on cellular metabolic processes, with alterations in proteins related to the electron transport chain, oxidative phosphorylation and ATP metabolism.

**Figure 5:**
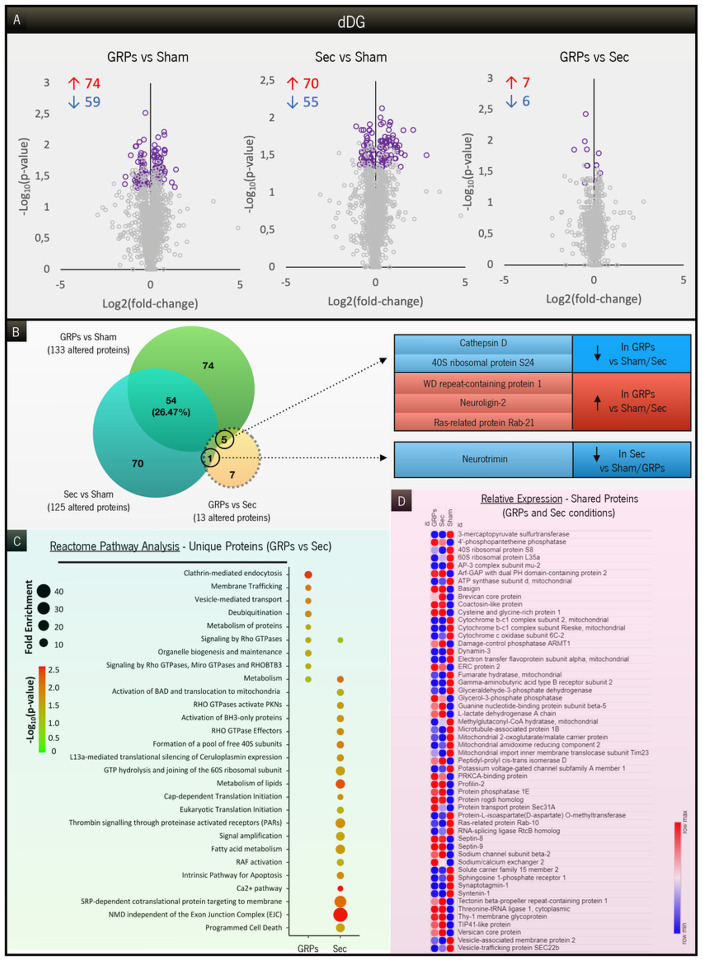
Analysis of the proteins altered between groups in the dDG region. (**A**) Volcano plots presenting the significantly altered proteins (highlighted in purple) in each comparison under study: GRPs vs. Sham, Sec vs. Sham, and GRPs vs. Sec. The number of increased (red) or decreased (blue) proteins is indicated for each comparison. (**B**) Venn diagram representing the specific number of proteins altered between GRPs vs. Sham, Sec vs. Sham, and GRPs vs. Sec. Six of the 13 proteins altered between treatments were also significantly altered compared to Sham animals, herein highlighted. (**C**) Reactome pathway analysis covering the most significant and enriched pathways, only considering the unique proteins in the comparisons of GRPs vs. Sham and Sec vs. Sham. (**D**) Heatmap of the relative expression levels of the 54 proteins commonly altered between GRP and secretome groups, in comparison to GFAP-tk animals.

**Figure 6:**
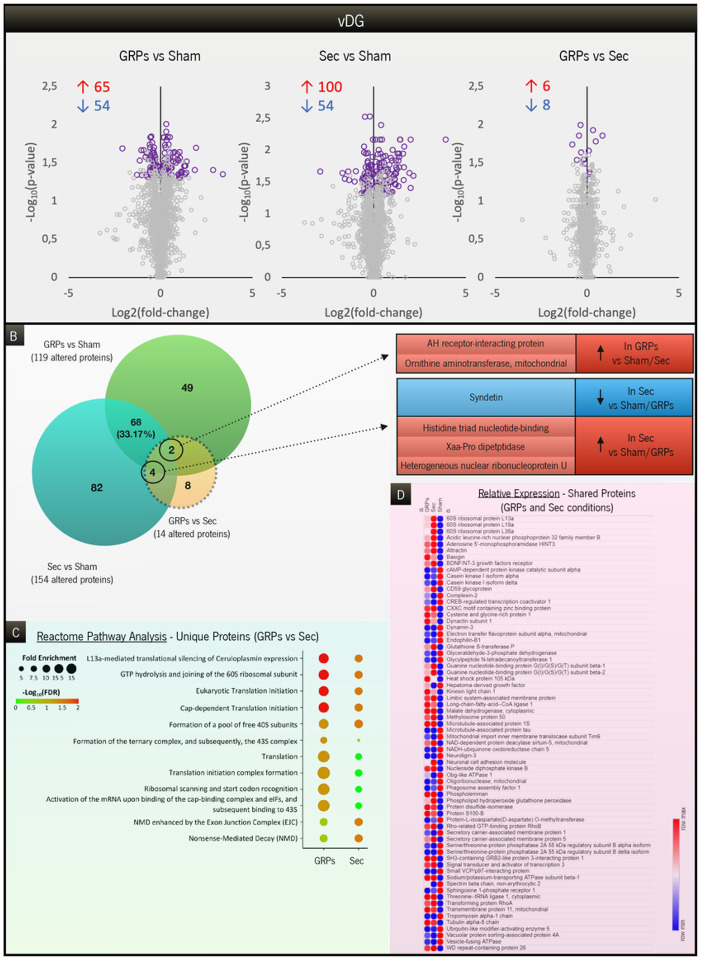
Analysis of the proteins altered between groups in the vDG region. (**A**) Significantly altered proteins (highlighted in purple), among the ones that were unique to each comparison under study: GRPs vs. Sham, Sec vs. Sham, and GRPs vs. Sec. The number of increased (red) or decreased (blue) proteins is indicated for each comparison. (**B**) Venn diagram representing the specific number of proteins altered between GRPs vs. Sham, Sec vs. Sham, and GRPs vs. Sec. Six of the 14 proteins altered between treatments were also significantly altered compared to Sham animals, herein highlighted. (**C**) Reactome pathway analysis covering the most significant and enriched pathways, only considering the unique proteins in the comparisons of GRPs vs. Sham and Sec vs. Sham. (**D**) Heatmap of the relative expression levels of the 68 proteins commonly altered between GRPs and Secretome groups, in comparison to GFAP-tk animals.

Next, we further dissected the results observed, either by addressing the proteins that were exclusively altered in each specific treatment (in comparison to GFAP-tk animals; Fig. 5A and 6A) or by looking at the ones that were changed in common (shared proteins; Fig. 5B and 6B). In the dDG (Fig. 5), the pool of unique proteins in the GRP group presented a completely different profile of reactome pathways, when compared to the one in the secretome group (Fig. 5C). In GRP-treated animals, the pathways involved in membrane trafficking and remodeling, metabolism of proteins and organelle biogenesis were enriched, whereas in the secretome-treated rats there was a specific enrichment in apoptotic-signaling pathways and protein synthesis. Interestingly, in the vDG (Fig. 6), the pools of unique proteins demonstrated a very similar profile of reactome pathways, focused mainly on protein synthesis processes (Fig. 6C), though with greater enrichment and significance in the GRP-treated group. Then, we compared relative expression levels for all the proteins commonly altered in both treatments in comparison to GFAP-tk animals, for both dDG and vDG (Figs. 5D and 6D, respectively). GRP-treated and secretome-treated rats presented a similar pattern of expression regarding commonly altered proteins when compared to GFAP-tk sham rats, with a few exceptions further highlighted in the Discussion section. Finally, we also found a restricted group of proteins that were significantly altered between GRP and secretome groups in both regions analyzed (Figs. 5B and 6B, for dDG and vDG, respectively). An increase in the levels of neuroligin-2 in the dDG (Fig. 5B) or of AH receptor-interacting protein in the vDG are examples of proteins that could help to elucidate the positive results observed in the GRP group, which are further addressed in the Discussion.

## Discussion

The generation of new cells in the human adult brain is still today a matter of intense debate (2, 5, 58). If this aspect remains contested, establishing the importance of newly born cells in overall brain networks and function is an even bigger challenge. In this context, our group and others have demonstrated that a defined group of cells (neural stem cells) continuously proliferate within restricted brain regions in adult rats (59, 60), and when those cells are specifically targeted, several behavioral domains are affected (37). However, most of the studies have been focused on the generation of new neurons, neglecting the relevance of newly born glial cells. In fact, just considering the higher proportion of glial cells in the mammalian brain, it is logical to foresee a critical role for newly generated glia. Furthermore, glial cells are involved with neurons in the arrangement of molecular signals that regulate the function of neuronal networks in the developing brain, possessing a fundamental role in regulating brain homeostasis. In this aspect, there is limited knowledge about the specific contribution of newly born glia to brain function, as well as a lack of information on their potential role in the development of new therapeutic routes for brain disorders. Hence, in this work, we aimed to specifically boost gliogenesis by introducing a population of glial progenitors (GRPs) into one of the most important cytogenic niches in the adult brain, the hippocampal DG, in a model in which the capacity of generating new cells was transiently compromised (37, 38)By doing so, we would be specifically increasing the generation of new glial cells, dissecting their role from the contribution of newly born neurons. This rationale was established based on recently published results (37), where we demonstrated that, following GCV administration, the GFAP-tk rat model presented a stable depletion of newly born cells for about two and half-weeks. Therefore, by transplanting GRPs or injecting their secreted factors within that time window, we assured a major contribution from transplanted glial progenitor cells (excluding the role of endogenous newborn cells).

The treatments were administered in the dDG and injections were confirmed to be given in the appropriate coordinates (Fig. 1E), as GRPs were mainly concentrated in the dDG (no cells were found in vDG sections), and no major cell migration patterns were observed. Additionally, we have not only confirmed that the transplanted cells survived (at least 40 days post-grafting), but they also proliferated and differentiated into glial-derived cells. These results go along with work from Hattiangady and colleagues in which GRPs transplanted into the hippocampus of aged animals remained clustered at the site of grafting, with minimal migration from the transplant core, and readily differentiated into astrocytes and oligodendrocytes (61). Then, regarding the induction of the GFAP-tk model, we have confirmed a significant suppression of BrdU + cells in the DG, which resulted in long-term pronounced anxiety- and depressive-like behaviors in transgenic animals. Interestingly, we have shown that a one-time transplantation of GRPs was capable of reverting not only these behavioral deficits, but also neurogenic and gliogenic levels, in particular in the vDG. Once again, these results are similar to the ones observed by Hattiangady and colleagues, in which GRP transplants elicited a boost of endogenous neurogenesis in aged WT rats (61). In our study, the administration of factors secreted by GRPs (8h conditioning protocol) was also sufficient to revert anxiety-like behaviors, but not depressive-like ones. Importantly, secretome-treated animals did not present significant alterations in neurogenesis or gliogenesis in comparison to GFAP-tk sham rats. These results indicate that transplanted glial progenitors boosted cytogenic levels in the vDG, and these new cells might have had a pivotal role in preventing the behavioral deficits characteristic of this animal model.

It is relevant to highlight that in this work, the GFAP-tk rat model presented depressive-like impairments, which contrasts with our previous findings using the same model (37). However, it should be stressed that in our previous results depressive-like behaviors were assessed at four weeks post-cytogenesis abrogation, while here the same tests (FST) were conducted between six-to-seven weeks after ending GCV treatment, which could account for the differences observed. In fact, Snyder et al. (62) have already shown that GFAP-tk mice present increased immobility times in the FST, indicative of a depressive-like phenotype. Apart from the animal model chosen, these mice were assessed 12 weeks post-cytogenesis ablation, once again accounting time as a decisive factor in the establishment of specific emotional deficits. Curiously, and as already mentioned, GRPs were transplanted into the dDG, with no APP + cells detected in vDG sections, suggesting that these two regions effectively communicate and are able to modulate each other. Traditionally, these regions have been regarded as functionally distinct, with the dorsal hippocampus being more associated with both learning and memory tasks (63–66), while the ventral hippocampus revealed to be a key structure of the emotional brain, responsible for regulating affective behaviors and modulating anxiety states (67–69). However, it has already been shown that both regions are in fact interdependent during spatial navigation tasks (64) or in novelty-induced contextual memory formation (70), corroborating the anatomical connections between them, which was already described decades ago (71–73). Hence, further studies are needed to understand this possible connection in the context of emotional behaviors, such as the ones affected in this work. Nevertheless, herein we have analyzed both regions individually, trying to dissect the impact of each treatment.

When analyzing all differentially expressed proteins, the pathways enriched in the dDG and vDG were very similar between GRP-treated and secretome-treated animals, with higher significance in the GRP-treated group, even though there are different players involved. No major differences were seen in the main clusters of biological processes (Fig. S2), with all regions analyzed (from both treatments) presenting at least one cluster related to neural processes, either neuronal communication or neurogenesis, among others. However, taking into consideration the proteins that were uniquely altered in each treatment, there is a clear difference in the profile of enriched proteins (and associated pathways) in the dDG. Furthermore, there is also a restricted group of proteins that were differentially expressed between GRP-treated and secretome-treated groups. For instance, neuroligin-2, which is enriched in the dDG of GRP-treated rats, is a transmembrane scaffolding protein involved in cell-cell communication via interaction with neurexin family members. In fact, several studies have already demonstrated the importance of this family of proteins for inhibitory synapse formation and function (74, 75), and the specific deletion of neuroligin-2 has resulted in anxiety-like behavior in mice (76, 77). This fits our data, where GRP-treated animals present higher levels of neuroligin-2 expression and, simultaneously, a recovery of the anxiety-like phenotype. Another protein specifically enriched in GRP-treated animals was the aryl hydrocarbon receptor-interacting protein, which was enhanced in the vDG. The aryl hydrocarbon receptor (AhR) is known to be present in nestin-expressing neural progenitor cells (78), and its experimental deletion adversely impacts neurogenesis (79). AhR-deficient mice have reduced cell birth, neuronal differentiation, and fewer mature neurons in the DG (79). Hence, its increased expression is logical, considering the augmented neurogenic and gliogenic levels registered in the vDG of GRP-treated rats. More recently, AhR has also been implicated in the regulation of granule neuron morphology and synaptic maturation (80). Finally, there was a group of proteins whose expression was enriched in both GRP and secretome groups compared to GFAP-tk sham animals. The expression levels of these proteins were not statistically different between GRP-treated and secretome-treated animals; however, this finding does not necessarily mean that biological significance could be disregarded. Notably, GRP-treated rats presented higher levels of the protein S100β in the vDG. Primarily expressed in astrocytes, it is logical that its expression is increased in regions with higher gliogenic levels. Moreover, and knowing that astrocytes can secrete S100β, it has been demonstrated that its intraventricular infusion enhanced hippocampal neurogenesis following traumatic brain injury (81). Another protein increased in the vDG was the heat shock protein HSP105, whose increased expression was previously associated with augmented hippocampal cell proliferation and recovery of depressive-like behavior in mice (82). Overall, some of these highlighted proteins might have played a role in the effects observed following GRP transplantation.

Nevertheless, the injection of the factors secreted by GRPs also resulted in the reversion of anxiety-related deficits in GFAP-tk rats. An unbiased reactome analysis focused on GRP secretome protein content revealed a myriad of different enriched pathways (Fig. S3A), from which positive regulation of neuron projection development and regulation of synapse organization can be highlighted. Furthermore, a total of 118 proteins related to neuronal development processes, neurogenesis and gliogenesis, regulation and behavioral processes were identified, possibly explaining the results observed (Fig. S3B). In fact, GRP secretome presented pro-cytogenic proteins such as nestin (83), fatty acid binding protein 7 (fabp7) (84) and brevican core protein (bcan) (85). In addition, other proteins associated with neuronal maturation, growth-associated protein 43 (gap43) (86) and neurocan core protein (cspg3) (87) were found. Proteins related to glial structure (GFAP) (88) and function [myelin-associated glycoprotein (Mag)] (89) were also detected in the proteomic analysis, which might be involved in gliogenesis. Considering this protein content, a protocol of repeated secretome injections, potentially through alternative routes of administration, would be interesting to study in future experiments.

The importance of newly born cells in health and disease is still far from being fully understood. To the best of our knowledge, this is one of the first studies to demonstrate that boosting levels of glial progenitors *in vivo* results in a faster recovery of cytogenic impairments in a transgenic rat model, thereby avoiding associated emotional deficits (anxiety and depressive-like behaviors). Transplanted cells elicited a cascade of events that resulted in increased cytogenic levels in the vDG, based on a mechanistic component that is yet to be identified. Further studies should aim to determine the exact role of transplanted glial progenitors and how they affect the communication between brain regions, such as the dorsal-ventral axis of the hippocampus. The genesis of mood disorders is indeed a result of delicate alterations in orchestrated brain neuronal and glial networks, to which more insights about the role of new neurons and new glia are of the utmost importance.
